# Supplementary material for: Hospital homebound students and K-12 online schooling
Source: PLoS One. 2022 Mar 24;17(3):e0264841. doi: 10.1371/journal.pone.0264841 (PMC8947101; doi:10.1371/journal.pone.0264841)
Supplement: Attachment — Submitted filename: revisionletter.doc [file pone.0264841.s001.doc]

**Department of Pediatrics**

**Division of General Pediatrics**

1600 SW Archer Road, H-5

PO Box 100291

Gainesville, FL 32611

Phone: 352-273-5322

To the Editor and Reviewers,

I am pleased to submit this revised manuscript, Hospital Homebound Students and K-12 Online Schooling, for review. I appreciate the insightful suggestions made by the editor and reviewers, we have worked to address each suggestion to the best of our ability. The authors received no specific funding for this work.

In response to a comment by the editor, we have included a more detailed explanation of data availability. Importantly, our ability to provide access to the data is limited by Florida law. Family Educational Rights and Privacy Act (FERPA), 20 U.S.C. § 1232g; 34 C.F.R Part 99; and chapter 119 and section 1002.22, Florida Statutes states: Individuals and organizations that receive data through the research request process have strict obligations to protect that data and must meet the requirements of both federal and state laws in their usage and handling of that data. The parties receiving this data have separate obligations based on these provisions and must protect the confidentiality and privacy of this information and may not disclose this information to others.

The Florida Department of Education requires all requests to comply with the Family Educational Rights and Privacy Act’s (FERPA’s) research exception (34 CFR Part 99.31[6][i]), which requires that the disclosure of information be limited to organizations conducting studies for, or on behalf of, educational agencies. In addition, the research must meet one of the following three allowable purposes: 1) to improve instruction; 2) to develop, validate or administer predictive tests; or 3) to administer student aid programs. Requests must meet one of these criteria to be approved.

In sum, the data is available via public request via Florida’s Government-in-the-Sunshine law. This provides the right of access to governmental documents at the state and local level. Data access can be facilitated using the Florida Department of Education External Research Data Request Process: <https://www.fldoe.org/accountability/accountability-reporting/external-research-requests/>. While this is not standard, we feel that this does not alter our adherence to  PLOS ONE policies on sharing data and materials.

I have attached a table that lists each suggestion, along with our response, inclusive of page numbers where the narrative addressing the response can be found.

Thank you for taking the time to reevaluate this submission.

Sincerely,

Erik W. Black, PhD, MPH

University of Florida

College of Medicine

[ewblack@ufl.edu](mailto:ewblack@ufl.edu)

352-273-5321

| 1. Please ensure that your manuscript meets PLOS ONE's style requirements, including those for file naming. The PLOS ONE style templates can be found at | | We have revised in accordance with style requirements. |
| --- | --- | --- |
| 2. In ethics statement in the manuscript and in the online submission form, please provide additional information about the records used in your retrospective study. Specifically, please ensure that you have discussed whether all data were fully anonymized before you accessed them and/or whether the IRB or ethics committee waived the requirement for informed consent. If patients provided informed written consent to have data from their medical or educational records used in research, please include this information. | | This has been addressed in the 1st paragraph of the Methods section and the first paragraph of the data and inclusion criteria subheading. |
| a) Please clarify the sources of funding (financial or material support) for your study. List the grants or organizations that supported your study, including funding received from your institution. | | These points have been addressed in our response to reviewers (to the best of our knowledge this is where the statement needed to be included). Specific veribiage requested in underlined in the response letter. Additional narrative has been added to the methods section. |
| b) State what role the funders took in the study. If the funders had no role in your study, please state: “The funders had no role in study design, data collection and analysis, decision to publish, or preparation of the manuscript.” | |  |
| c) If any authors received a salary from any of your funders, please state which authors and which funders. | |  |
| d) If you did not receive any funding for this study, please state: “The authors received no specific funding for this work.” | |  |
| Please confirm that this does not alter your adherence to all PLOS ONE policies on sharing data and materials, by including the following statement: "This does not alter our adherence to  PLOS ONE policies on sharing data and materials.” (as detailed online in our guide for authors http://journals.plos.org/plosone/s/competing-interests).  If there are restrictions on sharing of data and/or materials, please state these. Please note that we cannot proceed with consideration of your article until this information has been declared. | |  |
| Please include your updated Competing Interests statement in your cover letter; we will change the online submission form on your behalf. | |  |
| 5. In your Data Availability statement, you have not specified where the minimal data set underlying the results described in your manuscript can be found. PLOS defines a study's minimal data set as the underlying data used to reach the conclusions drawn in the manuscript and any additional data required to replicate the reported study findings in their entirety. All PLOS journals require that the minimal data set be made fully available. For more information about our data policy, please see http://journals.plos.org/plosone/s/data-availability. | |  |
| Upon re-submitting your revised manuscript, please upload your study’s minimal underlying data set as either Supporting Information files or to a stable, public repository and include the relevant URLs, DOIs, or accession numbers within your revised cover letter. For a list of acceptable repositories, please see http://journals.plos.org/plosone/s/data-availability#loc-recommended-repositories. Any potentially identifying patient information must be fully anonymized. | |  |
| Important: If there are ethical or legal restrictions to sharing your data publicly, please explain these restrictions in detail. Please see our guidelines for more information on what we consider unacceptable restrictions to publicly sharing data: http://journals.plos.org/plosone/s/data-availability#loc-unacceptable-data-access-restrictions. Note that it is not acceptable for the authors to be the sole named individuals responsible for ensuring data access. | |  |
| We will update your Data Availability statement to reflect the information you provide in your cover letter. | |  |
| 6. Please include your full ethics statement in the ‘Methods’ section of your manuscript file. In your statement, please include the full name of the IRB or ethics committee who approved or waived your study, as well as whether or not you obtained informed written or verbal consent. If consent was waived for your study, please include this information in your statement as well. | |  |
|  | |  |
| **Introduction:** | |  |
| The sentence: “Meeting the educational needs…” appears to miss some words at the end. I recognized some more typos while reading the manuscript. The authors should proof read the manuscript. | | Thank you, this has been addressed |
| The author may want to provide an overview or “the present study” section at the end of the introduction, which helps the reader to grasp an overview of what this study is about. | | Thank you, we have added to the final paragraph of the introduction (pg.5) |
|  |  | |
|  |  | |
|  |  | |
| **Methods:** | |  |
| The online school should be described in more detail. No information at all in provided on which courses are offered, for which age/grade level, which subjects, etc. It should also be noted whether students receive feedback or how students get learning materials assigned. At the current version of the manuscript, no information at all is provided on how an “online course” looks like or what is meant by and “online course”. | | We have included a more detailed description and screen shots. 1-4 paragraphs methods (p5-7, images on pg. 6) |
| Inclusion criteria: | |  |
| A separate inclusion criteria section which describes the inclusion process may help the reader to grasp the inclusion process of the two cohorts better. | | We have separated this out to provide a better detailing. Methods features a subheading Data and Inclusion Criteria (pg.8) with this information. |
| Data Analysis: | |  |
| The data analysis and the motivation behind the data analysis is not described at all. Currently, the manuscript lacks any description of why the analyzes were conducted. Why did the authors examine these analyses and not others? Or were other analyzes performed but did not reach significance and were thus not reported? The motivation of why the dependent/independent variables were selected should be noted. | | We have added information about motivations (pg 10, paragraph 1 and 2). |
| The statistical software the analyzes were carried out with should be noted. | | This has been included into the methods section, final paragraph, pg 10. |
| **Results:** | |  |
| The authors should provide more descriptive variables on the data. What was the mean number of courses students enrolled in (range of enrolled courses)? How long does one course take? How many sessions does one course include (range)? How many different courses exist in this online school? Were all courses analyzed? More information here is needed. | | We have included a more detailed description of FLVS 1-4 paragraphs of methods (p5-7) as well as emphasized language in the results to better inform the read of the composition of groups. Paragraph 1 and 2 of Results provides information on average course instances. |
| Online courses in general vary heavily and information on this is necessary to compare this study with others describing performance outcomes in online courses. | | This has been included as a limitation (pg 20), we have also included a table (table 1, pg 9) with the complete listing of courses that were incorporated into the study. |
| Non-significant findings cannot be interpreted as support for no differences between groups! The authors should note this and may want to carry out a Bayesian Analysis to support their point or compute a Bayes Factor for their findings. | | The interpretations have been revised to reflect this point. We love the ideas of a Baysian analysis, perhaps a follow up study? |
| **Discussion:** | |  |
| The authors analyzed whether students remained enrolled or not. They may want to hint towards recent attempts addressing this by means of survival analyzes. The lack of a systematic and persistent usage of online courses is one of the main problems of online courses and attempts on how to increase the enrollment for longer periods of time is one of the most challenging future questions in the field of online courses (Bacca-Acosta & Avila-Garzon, 2021; Grutzmacher et al., 2019; Plank et al., 2008; Spitzer et al., 2021; Villano et al., 2018). | | Thank you for this recommendation and the references, we have included verbiage in the discussion (final paragraph) |
| The authors note the boom in online learning courses around the world due to the COVID-19 pandemic. They may want to bolster this argument by adding some more references to it providing evidence that several online learning environments experienced a usage boom since march 2020 (Meeter, 2021; Spitzer et al., 2021; Tomasik et al., 2020; Velde et al., 2021). | | Thank you for these helpful references, they have been included in the first paragraph of the discussion. |
| The authors may want to note that they did not control for any behavioral variables in their analysis such as time on task, number of problem sets, or repetition attempts students may have used. Future research may investigate differences in usage patterns between the two cohorts. | | Thank you for this recommendation , we have included verbiage in the discussion (final paragraph) |
